# Transcriptomic Analysis of the Claudin Interactome in Malignant Pleural Mesothelioma: Evaluation of the Effect of Disease Phenotype, Asbestos Exposure, and CDKN2A Deletion Status

**DOI:** 10.3389/fphys.2017.00156

**Published:** 2017-03-21

**Authors:** Erasmia Rouka, Georgios D. Vavougios, Evgeniy I. Solenov, Konstantinos I. Gourgoulianis, Chrissi Hatzoglou, Sotirios G. Zarogiannis

**Affiliations:** ^1^Gradute Program in Primary Health Care, Faculty of Medicine, University of ThessalyLarissa, Greece; ^2^Department of Respiratory Medicine, University of Thessaly Medical SchoolLarissa, Greece; ^3^Institute of Cytology and Genetics of the Siberian Branch of the Russian Academy of SciencesNovosibirsk, Russia; ^4^Department of Physiology, Novosibirsk State UniversityNovosibirsk, Russia; ^5^Department of Physiology, Faculty of Medicine, University of ThessalyLarissa, Greece

**Keywords:** asbestos, CDKN2A, claudins, interactome, malignant mesothelioma, pleura, tight junctions, transcriptome

## Abstract

Malignant pleural mesothelioma (MPM) is a highly aggressive tumor primarily associated with asbestos exposure. Early detection of MPM is restricted by the long latency period until clinical presentation, the ineffectiveness of imaging techniques in early stage detection and the lack of non-invasive biomarkers with high sensitivity and specificity. In this study we used transcriptome data mining in order to determine which *CLAUDIN* (CLDN) genes are differentially expressed in MPM as compared to controls. Using the same approach we identified the interactome of the differentially expressed *CLDN* genes and assessed their expression profile. Subsequently, we evaluated the effect of tumor histology, asbestos exposure, *CDKN2A* deletion status, and gender on the gene expression level of the claudin interactome. We found that 5 out of 15 studied *CLDNs* (*4, 5, 8, 10, 15*) and 4 out of 27 available interactors (*S100B, SHBG, CDH5, CXCL8*) were differentially expressed in MPM specimens vs. healthy tissues. The genes encoding the CLDN-15 and S100B proteins present differences in their expression profile between the three histological subtypes of MPM. Moreover, *CLDN-15* is significantly under-expressed in the cohort of patients with previous history of asbestos exposure. *CLDN-15* was also found significantly underexpressed in patients lacking the *CDKN2A* gene. These results warrant the detailed *in vitro* investigation of the role of CDLN-15 in the pathobiology of MPM.

## Introduction

Malignant Pleural Mesothelioma (MPM) is a highly aggressive tumor primarily associated with exposure to asbestos. Due to the prolonged latent period between asbestos exposure and clinical presentation of the disease, the incidence of MPM has continued to rise across Europe even after the imposed restrictions on asbestos use (Jennings et al., 2014). Worldwide the incidence of MPM is increasing and it is expected to peak in the years 2015–2025 (Robinson and Lake, 2005). MPM diagnosis and treatment is challenging, therefore current research efforts are focused in biomarker discovery that would aid its early diagnosis, prognosis and therapeutic outcome prediction (Brims et al., 2013). Transcriptome studies combined with data mining techniques have provided new insights into the pathogenesis of the disease and have led to the identification of new candidate biomarkers with potential clinical value (Melaiu et al., 2012).

Claudins represent a 24-member family of tetraspan transmembrane proteins that contribute to the formation and the proper function of tight junctions (TJs) (Valle and Morin, 2010). They regulate the paracellular transport of ions and molecules in a size and charge sensitive manner (Gonzalez-Mariscal et al., 2010). A notable feature of claudins is that some members increase the paracellular permeability while others decrease it. More specifically, claudin-2, -7, -10, -15, and -16 increase the paracellular cation permeability in the Tight Junctions (TJ) whereas claudin-3-, -4, -5, -8, -11, -14, and -18 have a sealing function (Amasheh et al., 2002, 2005; Milatz et al., 2010; Soini, 2011). In addition to their contribution to the establishment of cellular polarity, the TJ proteins also participate in the regulation of cell differentiation and proliferation (Facchetti et al., 2007b; Lal-Nag and Morin, 2009; Soini, 2011).

The pleural mesothelial cells (PMCs) form a monolayer that expresses claudins in their TJs, although their exact role in the pleural physiology and pathophysiology is scarcely investigated (Apostolidou et al., 2012). Currently it is known that mesothelial TJ claudins contribute to the pleural membrane permeability, while during inflammation the expression levels of claudins change leading to increased pleural permeability (Markov et al., 2011; Markov and Amasheh, 2014). These data highlight the role of claudins and TJ related proteins in the context of pleural physiology and pathophysiology.

Several studies have highlighted the role of claudins in cancer since in several malignancies (lung, kidney, breast, stomach, instestine, pancreas, and others) their expression is deregulated (Osanai et al., 2017). Furthermore, accumulating data suggest roles for claudins in tumor development and progression as well as in signal transduction while in several cancers expression of claudins is associated with prognosis (Osanai et al., 2017). Claudins have also been studied in the context of MPM by means of immunohistochemistry (Kleinberg et al., 2007; Chaouche-Mazouni et al., 2015). In this aspect several changes in the expression of claudins have been documented (Ouban and Ahmed, 2010; Soini, 2011). Most studies so far have focused on the diagnostic value of claudins, notably claudins 1–7, in distinguishing MPM from other carcinomas. Claudin-4 has been shown to be highly effective in the differential diagnosis between MPM and metastatic carcinomas (Facchetti et al., 2007a,b; Ohta et al., 2013; Ordóñez, 2013; Jo et al., 2014). However, at the transcriptional level there is scarcity of information regarding the differential gene expression of claudins between healthy and MPM samples.

In this study we applied data mining and transcriptomic analysis in order to determine the CLAUDIN (*CLDN*) genes that are differentially expressed in MPM specimens as compared to healthy controls. Using the same approach we also probed for CLDN interactors and assessed their expression patterns so as to identify novel genes interacting with *CLDNs* that may contribute to the pathophysiology of MPM. Finally, we evaluated the effect of MPM histology, asbestos exposure, *CDKN2A* deletion status, and gender on the gene expression level of the claudin interactome.

## Materials and methods

### Identification of the differential gene expression profile of CLDNs in microarray data from MPM and control counterparts

We used gene expression data from an MPM study included in the Oncomine Research Premium Edition Cancer Microarray database (http://www.oncomine.org) in order to investigate the gene expression profile of *CLDNs* (Gordon et al., 2005; GEO Profiles: GDS 1220). We analyzed data for the 15 *CLDN* genes that were assessed in this study (*CLDN-1, -3, -4, -5, -6, -7, -8, -9, -10, -11, -14, -15, -16, -17, -18*) in order to detect their potential differential expression in MPM specimens as compared to healthy ones. The genes that were not assessed in GDS 1220, were *CLDNs* -2, -12, -19, -20, -22, -23, -24,-25. In order to ensure that the data were generated with the same methodology, we selected gene expression data from a single study (a study that from this point on will be referred as Gordon Mesothelioma study throughout the text). In this study the Affymetrix Human Genome U133A array was used assessing 12.624 genes. The raw data were downloaded from Oncomine in Excel format, and were scrutinized selecting only the ones referring to surgically excised samples excluding the ones referring to mesothelial (1 sample) and MPM cell lines (4 samples). Finally, in this study there were *n* = 40 MPM cases and *n* = 9 controls (*n* = 5 pleura and *n* = 4 lung samples). The gene expression data were log transformed, median centered per array, and the standard deviation was normalized to one per array as described previously (Rhodes et al., 2004). All values from the transformed data were downloaded from Oncomine during March 2013.

### Identification of the interactome of the differentially expressed claudins

The gene interaction network of the significantly differentially expressed *CLDNs* in MPM patients was constructed using Bio-grid (http://thebiogrid.org), ConsensusPathDB (http://cpdb.molgen.mpg.de/) and String 9.05 (http://string-db.org/) databases. The Bio-grid database is a repository of genetic and protein-protein interactions that are curated from the primary biomedical literature for all major model organism species (Chatr-Aryamontri et al., 2013). The ConsensusPathDB database integrates interaction networks including binary and complex protein-protein, genetic, metabolic, signaling, gene regulatory, and drug-target interactions as well as biochemical pathways (Kamburov et al., 2009, 2013). String 9.05 is a database of known and predicted protein interactions including both direct (physical), and indirect (functional) associations (Szklarczyk et al., 2011).

After querying for each differentially expressed *CLDN* in the above databases, we superimposed the results in order to remove duplicate or triplicate genes and created a list of unique genes (*List 1*) that comprised the interactome of the significantly over- and under- expressed *CLDNs* in MPM patients from the Gordon Mesothelioma study. The members of *List 1* are shown in Table 1, where their name along with their description is provided based on GeneCards query that was performed. GeneCards (http://www.genecards.org) is an integrated database of human genes that provides concise genomic related information, on all known and predicted human genes (Safran et al., 2003). The members of *List 1* were subject to the differential gene expression analysis in the microarray data of the Gordon Mesothelioma study so as to identify their profile.

**Table 1:** **Interactome of the gene networks of claudin 4, 5, 8, 10, and 15**.

**Gene symbol**	**Gene description**
VKORC1	Vitamin K epoxide reductase complex, subunit 1
S100B	S100 calcium binding protein B
EPHA2	EPH receptor A2
GEM	GTP binding protein overexpressed in skeletal muscle
SHBG	Sex hormone-binding globulin
UBC	Ubiquitin C
OCLN	Occludin
TJP1	Tight junction protein 1
TJP2	Tight junction protein 2
TJP3	Tight junction protein 3
INADL	InaD-like (Drosophila)
CDH5	Cadherin 5, type 2 (vascular endothelium)
MPDZ	Multiple PDZ domain protein
WNK4	WNK lysine deficient protein kinase 4
CLDN1	Claudin 1
CLDN2	Claudin 2
CLDN3	Claudin 3
CLDN6	Claudin 6
CLDN9	Claudin 9
CLDN11	Claudin 11
CLDN12	Claudin 12
CLDN14	Claudin 14
CLDN16	Claudin 16
CLDN17	Claudin 17
CLDN18	Claudin 18
CLDN19	Claudin 19
CLDN20	Claudin 20
CLDN22	Claudin 22
CLDN23	Claudin 23
CLDN24	Claudin 24
CLDN25	Claudin25
ESAM	Endothelial cell adhesion molecule
TACSTD2	Tumor-associated calcium signal transducer 2
CCDC155	Coiled-coil domain containing 155
SYNE4	Spectrin repeat containing, nuclear envelope family member 4
ATE1	Arginyltransferase 1
MARVELD3	Marvel domain containing 3
CXCL8	Chemokine (C-X-C Motif) ligand 8
ETV5	Ets variant 5
GRM5	Glutamate receptor, metabotropic 5
FGF1	Fibroplast growth factor 1

### Identification of the differential gene expression profile of CLDNs interactome in microarray data from MPM with respect to tumor histology, asbestos exposure, CDKN2A deletion status, and gender

We used gene expression data from the only MPM study included in the Oncomine Research Premium Edition Cancer Microarray database (http://www.oncomine.org) that compared gene expression levels in the three main phenotypes of MPM (epithelioid, biphasic, and sarcomatoid) in order to investigate the gene expression profile of the claudin interactome in relation to disease phenotype (Lopez-Rios et al., 2006; a study that from this point on will be referred as Lopez-Rios Mesothelioma study throughout the text). Furthermore, the same genes were analyzed based asbestos exposure (exposed/unexposed), *CDKN2A* deletion status (deletion/no deletion), and gender (male/female). Reported asbestos exposure history was extracted by the sample annotation information file provided in the supplementary materials of the Lopez-Rios Mesothelioma study provided in http://cbio.mskcc.org/public/Ladanyi_lab_mesothelioma_datasets/. This study included adequate microarray data from each histological subtype of MPM (*n* = 69 epithelioid, *n* = 10 sarcomatoid, *n* = 20 biphasic). We analyzed data only for those genes that were found to be over- or under-expressed in the samples of MPM patients in the Gordon Mesothelioma study. All values from the transformed data were downloaded from Oncomine during March 2013 following the same procedures as in the case of the Gordon Mesothelioma study. The levels of gene expression were subsequently arranged into strata with respect to asbestos exposure history (exposed/unexposed), *CDKN2A* deletion status (deletion/no deletion) and gender (male/female). Regarding asbestos exposure history, 58 patients reported asbestos exposure history, 29 patients reported non-exposure to asbestos, while for 12 patients this information was unknown and they were excluded from the sub-analysis concerning the effects of asbestos exposure. Regarding *CDKN2A* deletion status, homozygous deletion was present in 59 patients, in 29 patients it was not present while in 19 patients the information was not available thus they were excluded from the subanalysis.

### Statistical analysis

GraphPad Prism 5.0 was used for statistical analysis. The Kolmogorov–Smirnov normality test was used to assess the data distribution. Comparisons of gene expression between MPM and healthy specimens were performed with the un-paired *t*-test for parametric data and the Mann–Whitney *U*-test for non-parametric data. The Benjamini–Hochberg False Discovery Rate (FDR) was employed for multiple correction testing, which reports FDR (or *q*-value), in order to corroborate the validity of the results. The calculation of the *q* statistic was based on the formula given by Rhodes et al. (2004). The mean values of gene expression in the three histological subtypes of MPM were compared with the One-Way ANOVA with Tukey's multiple comparison test for parametric data and the Kruskal–Wallis test with Dunn's multiple comparison test for non-parametric data. Analysis with respect to asbestos exposure history (exposed/unexposed), *CDKN2A* deletion status (deletion/no deletion) and gender (male/female) was performed with the un-paired *t*-test and the Mann–Whitney *U-*test for parametric and non-parametric data respectively. Statistical significance was set at the *p* < 0.05 and *q* < 0.05 level.

## Results

### Identification of the differential gene expression profile of CLDNs in microarray data from MPM and control counterparts

The gene expression of *CLDN-15* was found to be significantly over-expressed in MPM specimens (*q* < 0.002) as compared to healthy tissues. On the other hand, *CLDNs -4, -5, -8*, and *-10* were significantly under-expressed compared to controls (*q* = 0.036, *q* = 0.001, *q* = 0.003, and *q* = 0.001, respectively). There was no significant difference in the gene expression of *CLDN -1, -3, -6, -7, -11, -14, -16*, and *-17*. The results are summarized in Table 2.

**Table 2:** **Claudins differentially expressed in MPM patients**.

**Gene ID**	***q*****-value**	**FC**
**UP-REGULATED**		
CLDN15	0.002	6.351
**DOWN-REGULATED**		
CLDN4	0.036	−2.291
CLDN5	0.001	−4.055
CLDN8	0.003	−3.090
CLDN10	0.001	−1.484

### Identification of the interactome of the differentially expressed CLDNs

In the analysis performed in the 3 databases as explained in the Section Materials and Methods, 41 genes were found to constitute the interaction network of the significantly differentially expressed *CLDNs*. The symbols and descriptions of those genes, as reported in the GeneCards database are presented in Table 1. Gene expression data for further analysis in the Gordon Mesothelioma study were available for 27 interactors (gene symbols: *VKORC1, S100B, EPHA2, GEM, SHBG, UBC, OCLN, TJP1-3, INADL, CDH5, MPDZ, CLDN-1, -3, -6, -9, -11, -14, -16, -17, -18, TACSTD2, CXCL8, ETV5, GRM5, FGF1*) while no gene expression data were available for 14 interactors (gene symbols: *WNK4, CLDN -2, -12, -19, -20, -22, -23, -24, -25, ESAM, CCDC155, SYNE4, ATE1, MARVELD3***)**.

### Identification of the differential gene expression profile of the interactome members of the significantly differentially expressed CLDNs

The gene expression of *S100B, SHBG, CDH5*, and *CXCL8* were significantly under-expressed compared to controls (*q* = 0.001, *q* = 0.004, *q* = 0.015, and *q* = 0.007, respectively). The results are summarized in Table 3.

**Table 3:** **Claudin interactome genes differentially expressed in MPM patients**.

**Gene ID**	***q*****-value**	**FC**
S100B	0.001	−4.675
SHBG	0.004	−1.848
CDH5	0.015	−2.926
CXCL8	0.007	−3.605

### Evaluation of the effect of disease phenotype, CDKN2A deletion status, asbestos exposure, and gender

**Figure 1:**
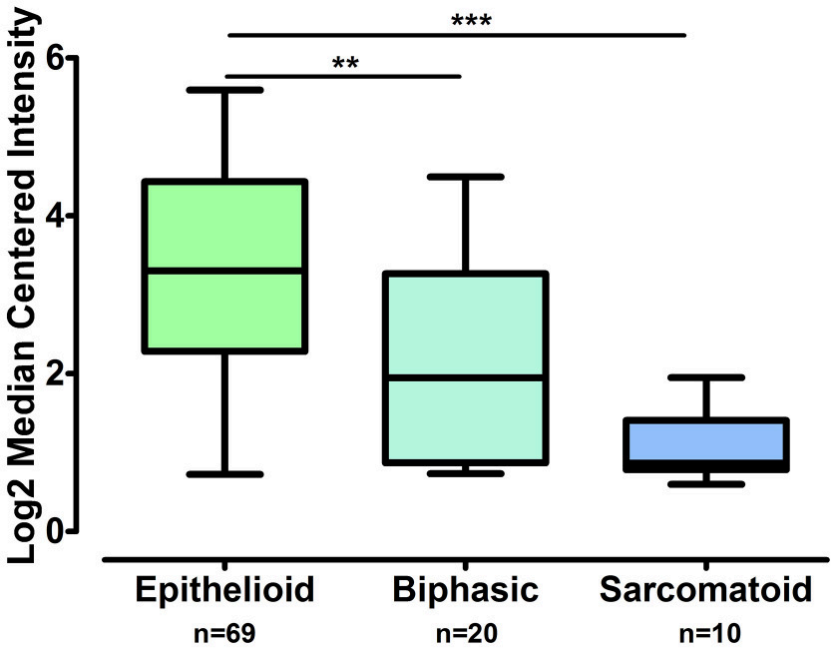
**CLDN-15 is over-expressed in epithelioid MPM as compared to biphasic and sarcomatoid MPM**. The number of patients per group is demonstrated in the figure. Gene expression of CLDN15 gene data was log transformed and normalized as described previously (Rhodes et al., 2004). ^**^p < 0.01; ^***^p < 0.001.

**Figure 2:**
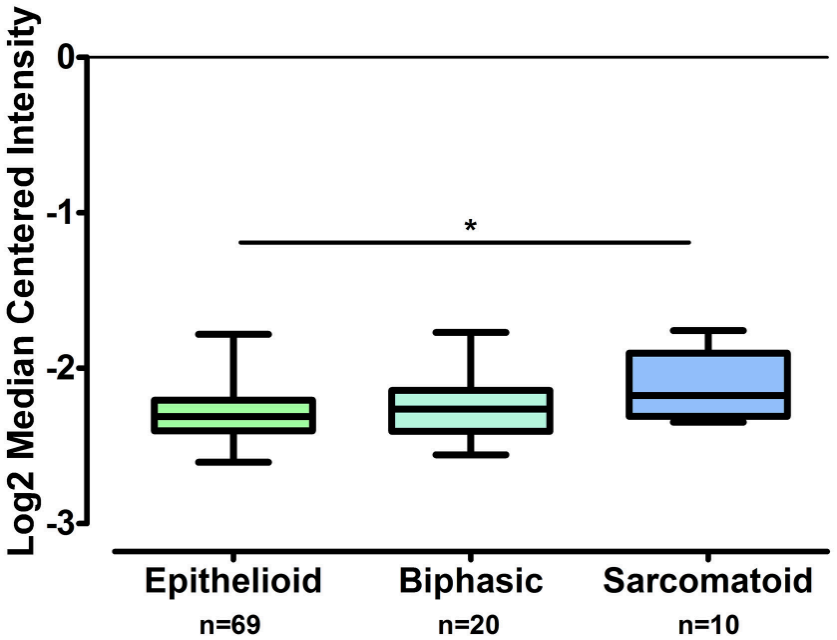
**S100B is over-expressed in sarcomatoid MPM as compared to epithelioid MPM while it does not differ from biphasic MPM**. The number of patients per group is demonstrated in the figure. Gene expression of S100B gene data was log transformed and normalized as described previously (Rhodes et al., 2004). ^*^p < 0.05.

**Figure 3:**
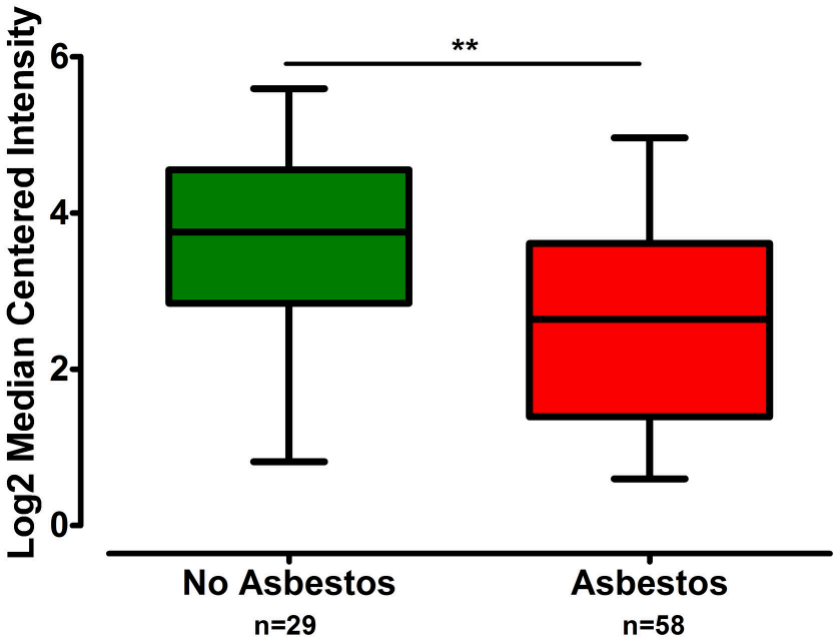
**CLDN-15 is under-expressed in MPM patients with history of asbestos exposure**. The number of patients per group is demonstrated in the figure. Gene expression of CLDN-15 gene data were log transformed and normalized as described previously (Rhodes et al., 2004). ^**^p < 0.01.

**Figure 4:**
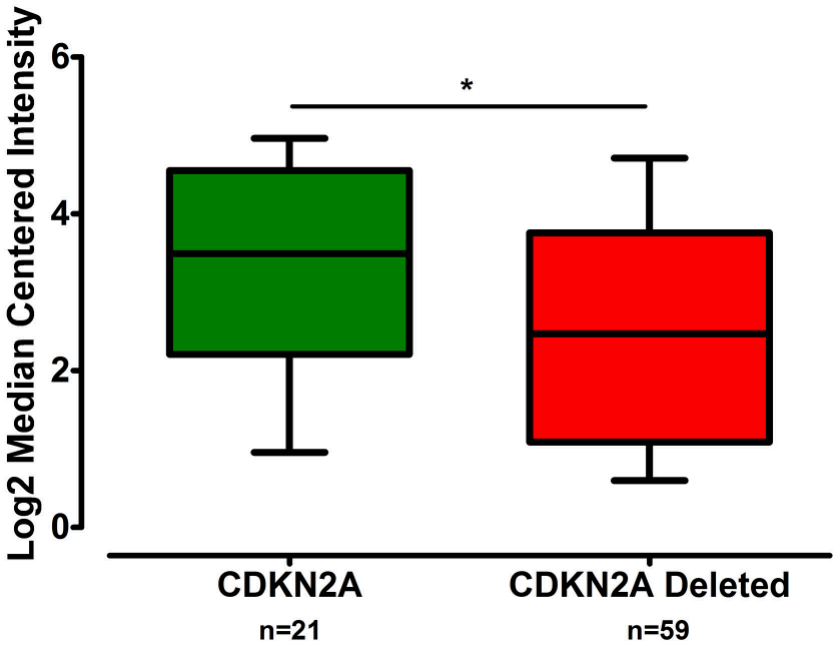
**CLDN-15 is under-expressed in MPM patients with deletion of the CDKN2A gene**. The number of patients per group is demonstrated in the figure. Gene expression of CLDN-15 gene data were log transformed and normalized as described previously (Rhodes et al., 2004). ^*^p < 0.05.

The gene expression of *CLDN-15* was found to be significantly increased in the epithelioid histological subtype of MPM as compared to both the biphasic and sarcomatoid phenotypes (*p* < 0.01 and *p* < 0.001, respectively; Figure 1). The *S100B* gene was found significantly over-expressed in the sarcomatoid type of MPM compared to the epithelioid one (*p* < 0.05; Figure 2). There was no significant difference in the gene expression of *CLDN-4, -5, -8, -10, SHBG, CDH5*, and *CXCL8* among the three histological subtypes of MPM. Subsequent analysis with respect to history of asbestos exposure showed that the *CLDN-15* gene was significantly under-expressed in the cohort of patients that were previously exposed to asbestos as compared to the unexposed ones (*p* = 0.004; Figure 3). In addition, *CLDN-15* was found significantly under-expressed in the subgroup of patients with homozygous deletion of the *CDKN2A* gene as opposed to the patients with no deletion (*p* = 0.035; Figure 4). Gene expression of *CLDN-4, -5, -8, -10, S100B, SHBG, CDH5*, and *CXCL8* was not influenced by asbestos exposure and CDKN2A deletion status. Finally, the gene expression of *CLDN-4, -5, -8, -10, -15, S100B, SHBG, CDH5*, and *CXCL8* was not influenced by gender.

## Discussion

MPM is a highly aggressive tumor arising from the mesothelial cells that line the pleural cavity (Rascoe et al., 2012). Due to the established cause and effect relationship between asbestos exposure and MPM development, researchers seek to identify suitable biomarkers for screening asbestos exposed populations and for early diagnosis while avoiding false positive results and unnecessary invasive procedures (Ostroff et al., 2012). Although, several studies have pointed to the effectiveness of soluble mesothelin as a biomarker with high specificity, its limited sensitivity confines its use as a screening tool for the asymptomatic, asbestos-exposed cohorts (Creaney et al., 2015). Thus, at the present time, there are no biomarkers in widespread clinical use for MPM (Creaney et al., 2015).

Transcript profiling along with new omics-technologies and bioinformatics, have been extensively used in the field of personalized medicine enabling researchers to identify biomarkers for cancer screening, diagnosis, and prognosis (Diamantis et al., 2010). In this study we used established data mining techniques through which we have already reported *AQP1, CLIC3, CLIC4*, and *BBS1* to be ideal candidates for further study in MPM pathophysiology (Jagirdar et al., 2013; Tasiopoulou et al., 2015; Vavougios et al., 2015).

Claudins are structural molecules of TJs present in epithelial, endothelial, and mesothelial cells (Soini et al., 2006; Soini, 2011). The patterns of claudin expression within TJs are distinctly diverse among different tissues accounting for the observed differences in permeability and electrical resistance of various epithelia (Chao et al., 2009; Markov et al., 2011). It should be noted that not only the expression of claudin family members but also their localization within cells may vary (Amasheh et al., 2002; Dittmann et al., 2014). The latter might be affected by several exogenous factors such as food components, plant compounds and microbial toxins (Dittmann et al., 2014; Markov et al., 2014).

Differential gene expression of claudins has been reported in several tumors depending on the exact claudin and cancer studied (Facchetti et al., 2007b; Chao et al., 2009; Lal-Nag and Morin, 2009; Gonzalez-Mariscal et al., 2010; Valle and Morin, 2010; Davidson, 2011; Soini, 2011). Here we report that *-15* is significantly over-expressed in MPM patients as compared to controls, while *CLDNs -4, -5*, and *-8* are significantly under-expressed. An *in vitro* study using M14K (epithelioid MPM) and M38K (biphasic MPM) cell lines (Chaouche-Mazouni et al., 2013) has shown by means of immunohistochemistry and immunoblotting a high level of claudin 15 in both cell lines as opposed to claudins 3 and 4. An earlier study demonstrated by means of immunohistochemistry that in MPM there was a lower expression of claudin-1, -3, -4, -5, and -7 than in metastatic adenocarcinomas of the pleura, suggesting that they could serve as differential diagnostic markers (Soini et al., 2006). In the same report, non-neoplastic PMCs showed expression of claudin 2 but no expression was found for claudin-3, -4, -5, and -7. Two more studies assessing the expression of claudin-4 in a large series of normal and MPM tissues by immunohistochemistry, reported that claudin-4 is a definitive negative marker for MPM which is in agreement with our data showing the significant under-expression of *CLDN-4* in MPM as opposed to controls (Facchetti et al., 2007a,b). This finding has been consistently shown in other studies as well (Ohta et al., 2013; Ordóñez, 2013; Jo et al., 2014). Finally one more study has demonstrated that the gene expression of *CLDN-3, -4*, and *-6* was significantly lower in malignant peritoneal mesothelioma as compared to ovarian carcinomas (Davidson et al., 2006).

In our study we also demonstrate that the *CLDN-15* gene is significantly over-expressed in the epithelioid histological subtype of MPM as compared to the sarcomatoid and biphasic phenotypes. Similar results were shown in diffuse malignant peritoneal mesothelioma at the protein level (Davidson et al., 2006).

On the contrary, we observed that the gene expression of *S100B* is significantly increased in sarcomatoid MPM as compared to epithelioid MPM. S100B has been reported as a useful biomarker in assessing tumor load, stage, and prognosis for patients with malignant melanoma (Zarogoulidis et al., 2015).

It has been suggested that the differences in the molecular biology of epithelioid and non-epithelioid MPM may contribute to differences in their clinical behavior (Balduyck et al., 2010). The epithelioid cell type is among the factors that favor the overall survival of MPM patients (Balduyck et al., 2010; Musk et al., 2011; Linton et al., 2014). As a consequence it has been proposed that the diagnosis of the sarcomatoid variant on a biopsy should preclude radical surgery, and therapy should aim at symptoms management and preservation of quality of life (Balduyck et al., 2010). Therefore, the clear distinction of histological subtypes could direct clinicians to the appropriate treatment options. In the literature it is supported that the current golden standard for MPM diagnosis is a combination of two positive and two negative immunohistochemical markers in the epithelioid and biphasic type, but sarcomatous type do not have specific markers, making diagnosis more difficult (Panou et al., 2015). Here we propose that *CLDN-15* and *S100B* could serve as complementary diagnostic tools and should be further investigated.

Next we observed that *CLDN-15* was under-expressed in patients lacking the *CDKN2A* gene. Moreover, the *CLDN-15* gene was significantly under-expressed in MPM patients with previous history of asbestos exposure compared to the unexposed ones. *CDKN2A* is the most frequently inactivated tumor suppressor gene in human MPM. The inactivation of both p16^INK4a^ and Arf products of the *CDKN2A* gene has been suggested to act synergistically in accelerating asbestos-induced tumorigenesis *in vivo* (Sekido, 2013). Development of MPM is strongly associated with asbestos exposure, with 80% of the patients having previous exposure to asbestos fibers (Robinson and Lake, 2005; Nakano, 2008; Sekido, 2013). A significant association has been described between increasing number of methylated cell cycle control genes and asbestos burden. This finding was followed by the observation that quantitative measure of asbestos exposure was associated with over 100 discrete CpG loci and that in 94% of cases there was increased methylation associated with increased exposure (Christensen and Marsit, 2011). The correlation between methylation status and extended exposure to asbestos was also confirmed by Fujii et al. (2012). Likewise, the results of this study are indicative of the epigenetic effect of asbestos in the transcriptional level of the *CLDN-15* gene suggesting its potential utility as a screening biomarker for populations at risk.

Although, in our analysis with respect to gender the gene expression of *CLDN-4, -5, -8, -15, S100B, SHBG, CDH5*, and *CXCL8* was not different between sexes, we observed that the *SHBG* gene which encodes the Sex Hormone-Binding Globulin was under-expressed in MPM patients as compared to healthy controls. *SHBG* is a major regulator of free plasma androgens and also mediates androgen and estrogen signaling at the cell membrane via cyclic adenosine monophosphate (Mononen and Schleutker, 2009).

It is generally accepted that although MPM is less common in women compared to men, female MPM patients survive longer (Balduyck et al., 2010; Musk et al., 2011; Linton et al., 2014; Taioli et al., 2014). It has been proposed that differences in asbestos exposure, tumor biology, and the impact of circulating hormones on host response must be investigated to understand this survival advantage and improve prognosis for patients of both genders (Taioli et al., 2014). The observed down-regulation of the *SHBG* gene in the group of MPM patients relative to the control group advocates the role of sex hormones in the pathogenesis and prognosis of the disease. This is stressed out by a study showing that estrogen receptor beta (ERβ) acts as a tumor suppressor of high potential relevance to prediction of disease progression and to therapeutic response in MPM patients (Pinton et al., 2009).

As in the case of *SHBG*, the genes encoding S100B, CDH5 and CXCL8 were found significantly under-expressed in MPM specimens as compared to their healthy counterparts. The protein product of *S100B* interacts with its target proteins within cells to regulate enzyme activities, cell growth, differentiation and Ca^2+^ homeostasis (Pang et al., 2012). It is thought that expression levels of individual S100 family proteins vary considerably in different tumors and with respect to cancer progression (Harpio and Einarsson, 2004).

CXCL8 or interleukin-8 (IL-8) is a proinflammatory CXC-type chemokine involved in the promotion of neutrophil chemotaxis and degranulation (Waugh and Wilson, 2008). It has been demonstrated that many types of human carcinomas express high levels of IL-8 relative to normal tissues (Palena et al., 2012). In addition, high serum levels of this chemokine correlate with disease progression and poor prognosis while a link exists between IL-8, tumor epithelial-mesenchymal transition (EMT) and tumor stemness (Palena et al., 2012; Gales et al., 2013). In this study, *IL-8* gene was found down-regulated in the group of MPM patients compared to the control group. It has been demonstrated that neutrophil infiltration into the pleural space is a characteristic feature of an early and acute inflammatory response in several pleural diseases and that activated PMCs secrete IL-8 in a polarized fashion (Nasreen et al., 2001). Inhibition of IL-8 has been shown to reduce human MPM propagation in a nude mouse model (Galffy et al., 1999). Asbestos directly stimulate PMCs to synthesize IL-8, possibly playing an important role in mediating asbestos induced pleural inflammation (Batra and Antony, 2015; Mutsaers et al., 2015). Further studies are warranted to compare IL-8 expression and features of EMT at various stages of tumor development (Palena et al., 2012).

Vascular endothelial cadherin (VE-cadherin; CDH5), an endothelial specific cell-cell adhesion molecule, plays a pivotal role in the formation, maturation, and remodeling of the vascular wall (Gavard, 2009). It has been shown that CDH5 directly enhances the expression level of CLDN-5 by tethering repressive transcription factors away from the CLDN*-5* promoter. Conversely, the absence of functional CDH5 is associated with loss of CLDN-5 expression (Taddei et al., 2008; Gavard, 2009). These results suggest that any changes in CDH5 will impact the endothelial barrier function at multiple levels and also explain why CDH5 inhibition may cause a marked increase in permeability (Gavard and Gutkind, 2008; Taddei et al., 2008). Increased permeability is an early step in the angiogenic process enabling endothelial migration out of the primary vessel in order to begin formation of the tumor neovasculature (Le Guelte et al., 2011). On the other hand, it has been demonstrated that induction of CDH5 during EMT promotes breast cancer progression via TGFβ signaling indicating that in certain tumor cells, CDH5 can induce cellular responses that are in contrast to its role in cell-cell contact growth inhibition in endothelial cells (Labelle et al., 2008). Thus, there are two distinct functions of CDH5 both in angiogenesis and progression of cancer (Labelle et al., 2008).

Regarding thoracic tumors in particular, it has been reported that VE-cadherin may be an interesting marker for analysis in anti-angiogenic therapeutic trials although results from clinical studies are pending (Reinmuth et al., 2010).

An important limitation of our findings is the lack of information regarding the asbestos exposure history of the MPM patients of the Gordon Mesothelioma study. If these data were available we could assess asbestos induced gene expression changes between MPM patients and controls. Our results require further investigation in the clinical setting with the inclusion of a large series of MPM patients vs. healthy asbestos exposed individuals serving as controls. Moreover, detailed information regarding the duration of asbestos exposure and the type of exposure (environmental/occupational) should also be incorporated in future research.

## Conclusions

The results of this study are suggestive of a distinct gene expression profile of the claudin interactome in MPM and underline the epigenetic effect of asbestos on the transcriptional level of the *CLDN-15* gene. The role of claudin-15 and S100B in pleural physiology and pathophysiology is unknown and requires in depth investigation at the functional level. Additionally, both *CLDN-15* and *S100B* should be further investigated as gene biomarkers in MPM with respect to their potential to discriminate the histological MPM subgroups at the molecular level and thus provide tools for personalized therapy.

## Author contributions

ER contributed in the study design; acquired, analyzed, and interpreted data; drafted the manuscript; gave final approval of the submitted version; agreed to be accountable for all aspects of the work. GV contributed in data analysis and interpretation; revised the draft critically; gave final approval of the submitted version; agreeded to be accountable for all aspects of the work. ES contributed in data interpretation; revised the draft critically; gave final approval of the submitted version; agreeded to be accountable for all aspects of the work. KG contributed in data interpretation; revised the draft critically; gave final approval of the submitted version; agreeded to be accountable for all aspects of the work. CH contributed in data interpretation; revised the draft critically; gave final approval of the submitted version; agreeded to be accountable for all aspects of the work. SZ conceived and designed the study; acquired, analyzed, and interpreted data; revised the draft critically; gave final approval of the submitted version; agreed to be accountable for all aspects of the work.

## Funding

This work was funded by the Postgraduate Program in Primary Health Care by the Faculty of Medicine of the University of Thessaly, BIOPOLIS, Larissa, Greece.

### Conflict of interest statement

The authors declare that the research was conducted in the absence of any commercial or financial relationships that could be construed as a potential conflict of interest.
